# A citrus fruits and leaves dataset for detection and classification of citrus diseases through machine learning

**DOI:** 10.1016/j.dib.2019.104340

**Published:** 2019-08-22

**Authors:** Hafiz Tayyab Rauf, Basharat Ali Saleem, M. Ikram Ullah Lali, Muhammad Attique Khan, Muhammad Sharif, Syed Ahmad Chan Bukhari

**Affiliations:** a Department of Computer Science, University of Gujrat, Gujrat, Pakistan; b Department of Agriculture, Jail Road Sargodha, Punjab, Pakistan; c Department of Computer Science & Engineering, HITEC University, Museum Road, Taxila, Pakistan; d Department of Computer Science, COMSATS Institute of Information Technology Wah Campus, Pakistan; e Division of Computer Science, Mathematics and Science, College of Professional Studies, St. John's University, New York, USA

**Keywords:** Image classification, Feature extraction, Feature selection

## Abstract

Plants are as vulnerable by diseases as animals. Citrus is a major plant grown mainly in the tropical areas of the world due to its richness in vitamin C and other important nutrients. The production of the citrus fruit has been widely affected by citrus diseases which ultimately degrades the fruit quality and causes financial loss to the growers. During the past decade, image processing and computer vision methods have been broadly adopted for the detection and classification of plant diseases. Early detection of diseases in citrus plants helps in preventing them to spread in the orchards which minimize the financial loss to the farmers. In this article, an image dataset citrus fruits, leaves, and stem is presented. The dataset holds citrus fruits and leaves images of healthy and infected plants with diseases such as Black spot, Canker, Scab, Greening, and Melanose. Most of the images were captured in December from the Orchards in Sargodha region of Pakistan when the fruit was about to ripen and maximum diseases were found on citrus plants. The dataset is hosted by the Department of Computer Science, University of Gujrat and acquired under the mutual cooperation of the University of Gujrat and the Citrus Research Center, Government of Punjab, Pakistan. The dataset would potentially be helpful to researchers who use machine learning and computer vision algorithms to develop computer applications to help farmers in early detection of plant diseases. The dataset is freely available at https://data.mendeley.com/datasets/3f83gxmv57/2.

**Table:** Specifications Table

Subject	Computer Science
Specific subject area	Image identification, Image classification, Image processing and computer vision
Type of data	Images
How data were acquired	Images are captured by using a single camera.
Data format	JPG, Raw
Parameters for data collection	Healthy and infected images of citrus fruits and leaves were collected separately. The infected images are further subcategorized into the disease, each citrus plant holds i.e Black spot, Canker, Scab, Greening and Melanose.
Description of data collection	No such sample pre-treatment was conducted. The data is collected manually with the help of domain expert and Citrus Research Center, Government of Punjab, Pakistan.
Data source location	**Institution**: University of Gujrat.
	**City**: Sargodha
	**Country**: Pakistan
Data accessibility	Dataset can be accessible at Mendeley data: https://data.mendeley.com/datasets/3f83gxmv57/2
Related research article	**Article 1:**
	**Author's name:** Sharif, M., Khan, M. A., Iqbal, Z., Azam, M. F., Lali, M. I. U., & Javed, M. Y.
	**Title:** Detection and classification of citrus diseases in agriculture based on optimized weighted segmentation and feature selection.
	**Journal:***Computers and electronics in agriculture*
	**DOI:**https://doi.org/10.1016/j.compag.2018.04.023
	**Article 2:**
	**Author's name:** Ali, H., Lali, M. I., Nawaz, M. Z., Sharif, M., & Saleem, B. A.
	**Title:** Symptom based automated detection of citrus diseases using color histogram and textural descriptors.
	**Journal:***Computers and electronics in agriculture*
	**DOI:**https://doi.org/10.1016/j.compag.2017.04.008

**Table:** 

**Value of the data** • This dataset provides visual tracking of citrus plant diseases in a hyperspectral sequence of citrus plants images. Therefore, it enables researchers to perform chemometric examinations for early identification of disease in citrus plant trees. • The dataset could be useful to test and compare different computer vision and image processing classifiers for the detection of different diseases based on their visual features. • Supports numerous feature selector and feature extractor by their textural descriptors and color scheme of different types of citrus diseases. • Original citrus plant images are taken from broader view (at leaf level), so could be desirable by the plant physiologist for the depth analysis. • The symptoms exerted from the dataset will facilitate comparison of the local citrus plants diseases structure with the other species of citrus plants located in Africa and surroundings. • This data is collected in a natural domain with inconsistent weather and light intensity. Hence there could be a challenge for the researchers to identify the disease symptoms with naked eye.

## 1 Data

Fruit plants play a significant role in the economic growth of any state. One of the famous species among the fruit plants is a citrus plant, which is full of vitamin C and broadly used in the region of the Middle East and Africa [1]. In the agro-industries, some kind of citrus plants are used as a raw material [2]. Citrus plant diseases is a major cause to reduce the production of citrus fruits and their usages in several industries. The most common disease identified by the domain experts and researchers are Greening, Melanose, Downy, Black spot, Canker, Scab, and Anthracnose. These citrus plants disease can be identified on the basis of their visual symptoms by applying some computer vision and deep learning techniques [3]. This article presents the data set contain 759 images of healthy and unhealthy citrus fruits and leaves that could be used by the researchers to apply different computer vision and image processing algorithm for the detection of citrus plants diseases. The data set is associated with the articles [4], [5]. All images were acquired manually using a DSLR with the help of domain expert of citrus disease and from the Sargodha region, Pakistan. The width and height for all images with the resolution of 72 dpi were 256 pixels and 256 pixels respectively.

The infected images were classified into 4 different diseases of citrus fruits and leave separately. The disease we targeted in the data sets were Black spot, Canker, Scab, Greening, and Melanose. Table 1 contains the description of the data set against each of its disease class. The dark spot on the citrus plants likewise shortened as CBS is accessible at the period when the plant is sick and the climate is ideal for the disease. The indications of the citrus leaf and fruits are little, bowed, and under hazardous spots with dark squares. The spectral range for spot diameter is 0.12–0.4 [4]. The canker spot is covered by the wind-driven downpour. On the citrus leaf, the small circles appear and the scope of the lesion is 2–10mm in size. Where the minimum range of citrus fruit lesion is appeared to be 1–10mm in size and these ranges contrast in size from one another [6]. Similarly the fruit species of citrus plants, the scab skin break out is a composite of parasitic and creature tissue. These acnes are not raised and the color shades vary from pink to light brown [6].

**Table 1:** Description of data set against each of its disease class.

Citrus Leaves		Citrus Fruits	
Disease	Number of Images	Disease	Number of Images
Black Spot	171	Black Spot	19
Canker	163	Canker	78
Greening	204	Greening	16
Melanose	13	Scab	15
Healthy	58	Healthy	22
Total Images	**609**	Total Images	**150**

Bold represents the total number of disease images captured in citrus leaves and fruits.

**Fig. 1:**
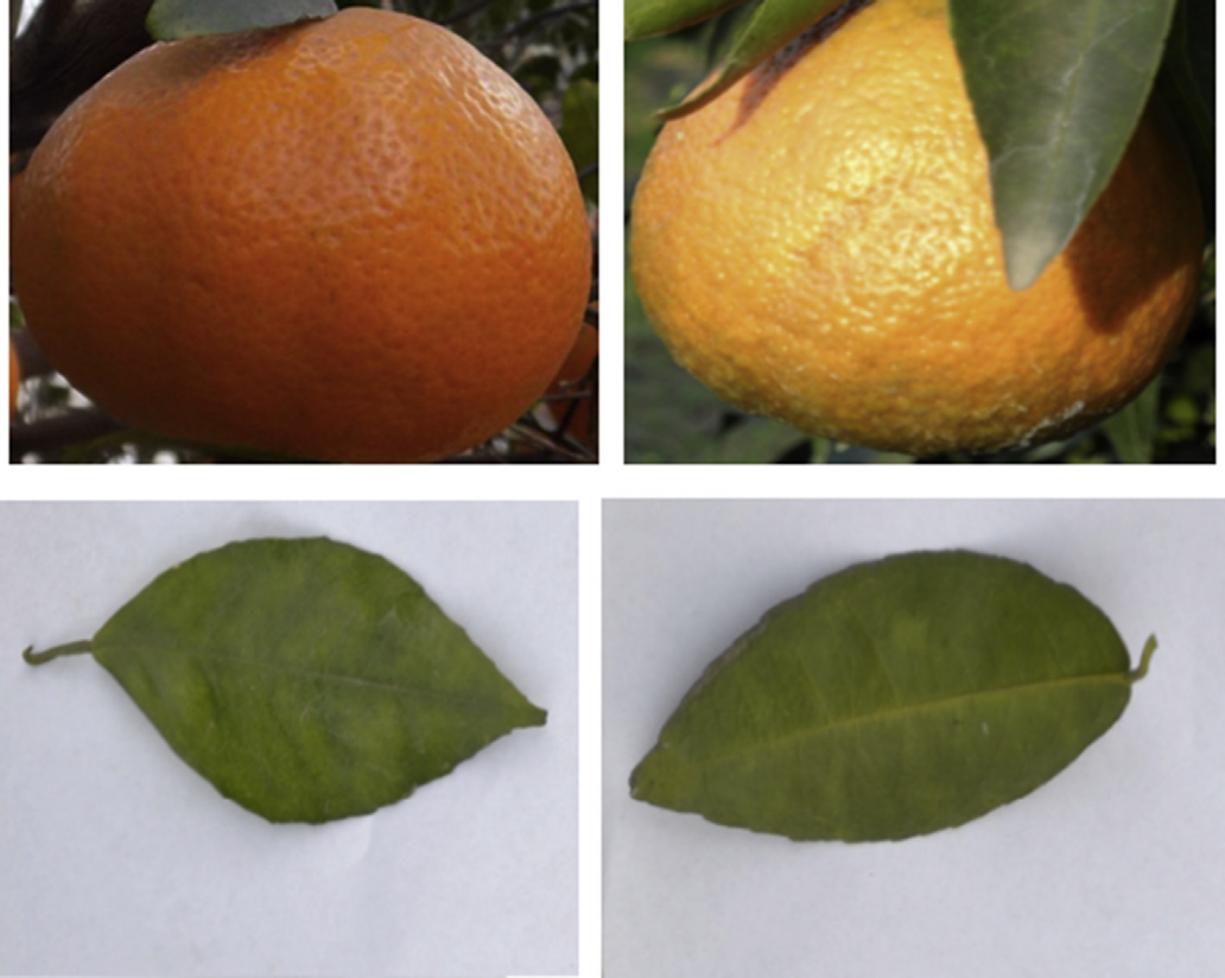
Samples of self-annotated images of healthy citrus fruits and leaves taken from the own collected dataset.

**Fig. 2:**
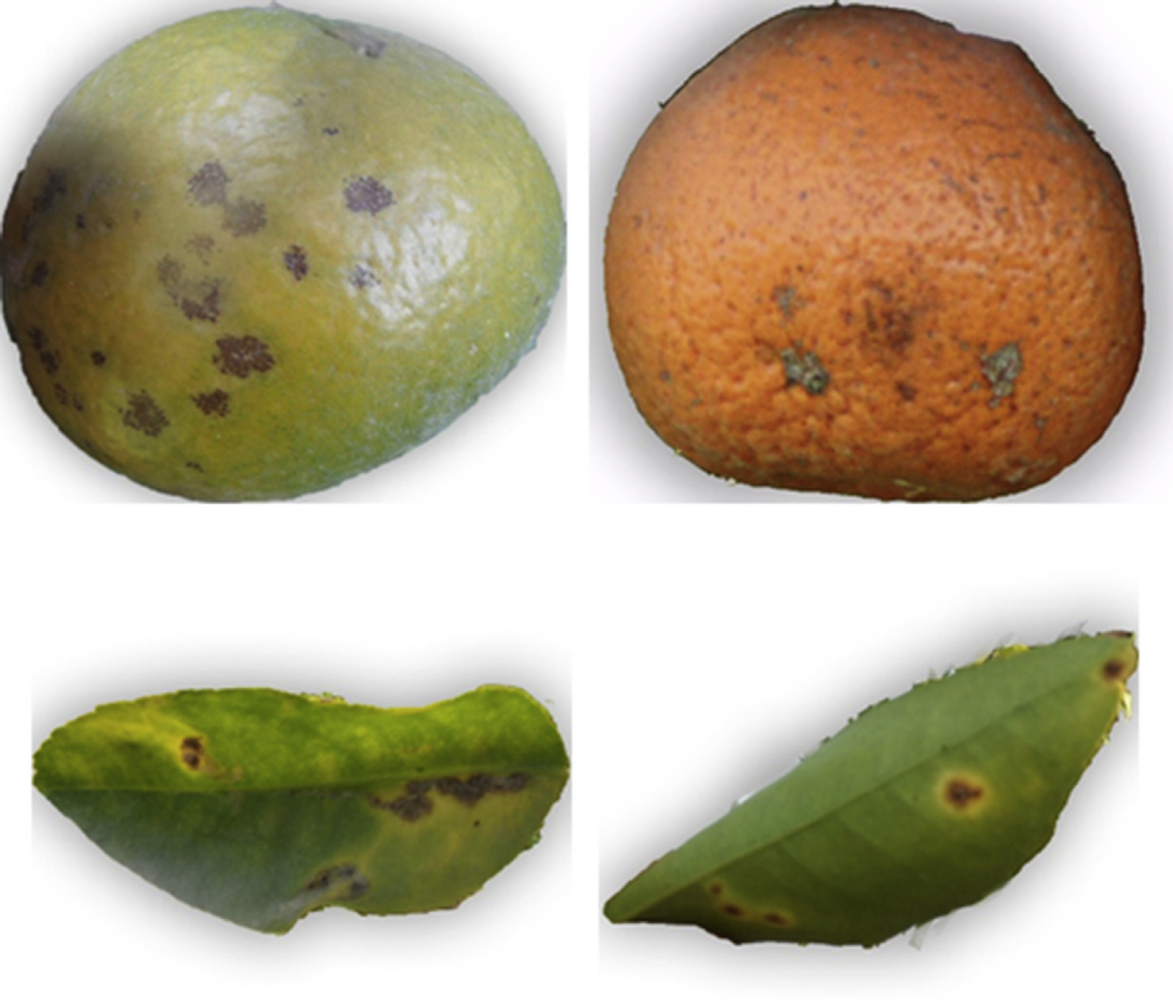
Samples of self-annotated images of citrus leaves and fruit with Black Spot diseases taken from the own collected dataset.

**Fig. 3:**
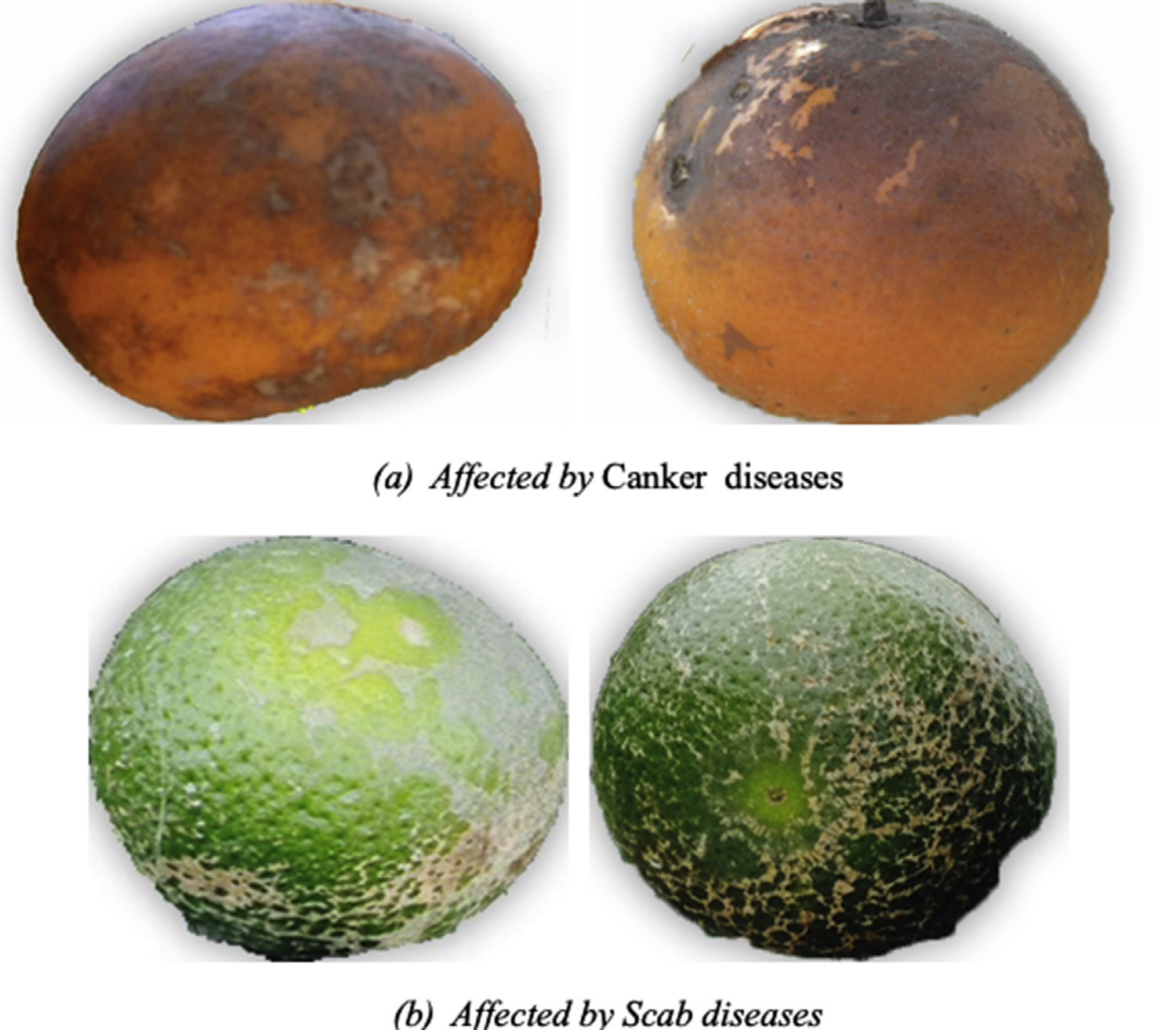
Samples of self-annotated images of citrus fruit with a) Canker b) Scab taken from the own collected dataset.

**Fig. 4:**
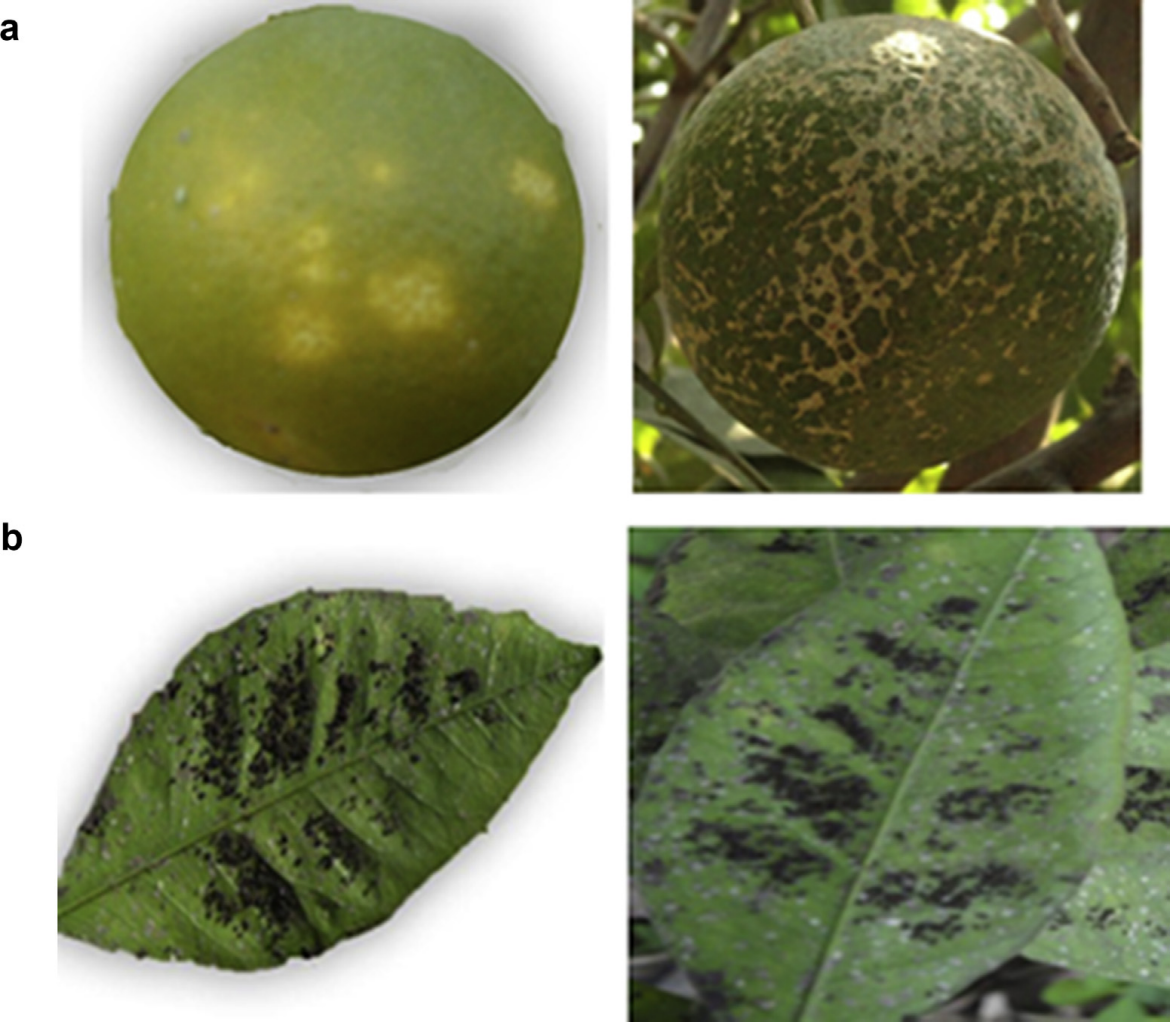
Samples of self-annotated images of citrus fruit and leaves with a) Greening and b) Melanose diseases taken from the own collected dataset.

Melanose is referred to as saprophyte where the weight of disease is characterized by total units of inoculum that make an influence on dead wood in the tree top. The sign of the disease is showing up as a little darker spot which ends up stuffed with a rosy dark colored gum. However, the indications of the disease on fruit species rely upon the age of the organic product at disease time [4]. Fig. 1, Fig. 2, Fig. 3, Fig. 4 showing the self-annotated images of healthy and un-healthy citrus fruit and plants.

## 2 Experimental design, materials, and methods

### 2.1 Camera specification

The data set were gathered using an advanced DSLR (Canon EOS 1300D) having a sensor with CMOS system and resolution of 5202 × 3465 (Mpix). The sensor size for Canon EOS 1300D is 14.9 × 22.3 (mm). RGB color range is chosen for each of the images in the JPG combination, including 256 shades for each RGB layer and 8 pixels for each shading layer.

### 2.2 Processing

**Fig. 5:**
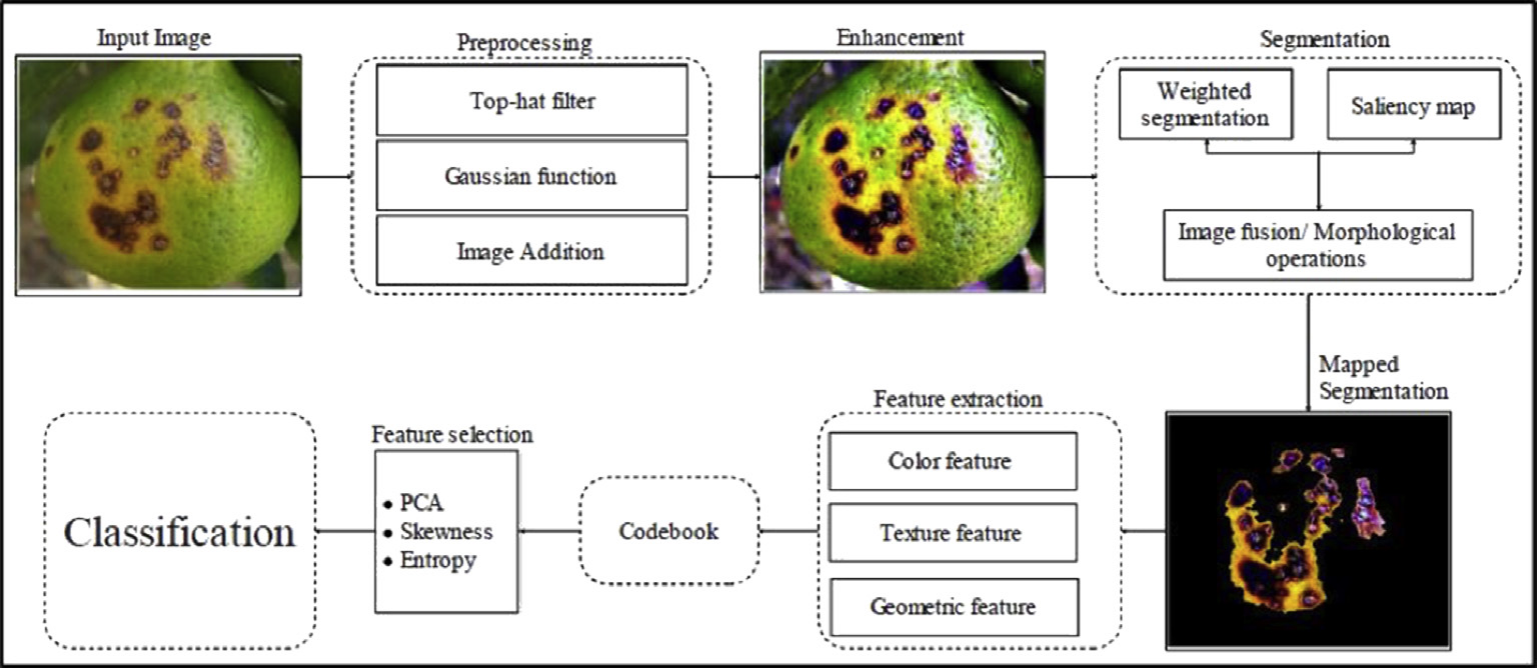
System architecture for identification and classification of infected plants diseases directly taken from Ref. [4].

All images are resized to the dimension of 256*256 with 72 dpi resolution. In Ref. [4], the presented data set is used by the authors for the detection and classification of citrus diseases. The entire processes include four major steps to complete which are: (a) enhancing the dataset, (b) lesion segmentation which highlights the infected region, (c) extracting features from the infected region and finally (d) selecting feature visually and perform classification. Firstly, all original input images were filtered by the Top-hat process and then Gaussian function is applied to improve the contrast of the infected region. Secondly, the enhanced images then mapped to the segmented blocks by implementing the weighted segmentation and Saliency map. At this stage, the input data set is segmented with lesion spots identified. Afterward, the segmented images were given to feature extractor and selector algorithms which extract texture, color, and geometric features and select those features using skewness, PCA, and Entropy methods. Finally, the classification is performed to classify each image instance corresponds to each of its disease class. Each process is made out of the composition of steps as displayed in Fig. 5.
